# Dual imaging stress echocardiography versus computed tomography coronary angiography for risk stratification of patients with chest pain of unknown origin

**DOI:** 10.1186/s12947-015-0013-8

**Published:** 2015-04-21

**Authors:** Quirino Ciampi, Fausto Rigo, Elisabetta Grolla, Eugenio Picano, Lauro Cortigiani

**Affiliations:** Division of Cardiology, Fatebenefratelli Hospital, Viale Principe di Napoli, 12 I-82100, Benevento, Italy; CNR Institute of Clinical Physiology, Pisa, Italy; Department of Cardiology, dell’Angelo Hospital, Mestre-Venice, Italy; Division of Cardiology, Lucca Hospital, Lucca, Italy

**Keywords:** Coronary flow reserve, Computed tomography coronary angiography, Coronary artery disease

## Abstract

**Background:**

Dual imaging stress echocardiography, combining the evaluation of wall motion and coronary flow reserve (CFR) on the left anterior descending artery (LAD), and computed tomography coronary angiography (CTCA) are established techniques for assessing prognosis in chest pain patients. In this study we compared the prognostic value of the two methods in a cohort of patients with chest pain having suspected coronary artery disease (CAD).

**Methods:**

A total of 131 patients (76 men; age 68 ± 9 years) with chest pain of unknown origin underwent dipyridamole (up to 0.84 mg/kg over 6 min) stress echo with CFR assessment of LAD by Doppler and CTCA. A CFR ≤ 1.9 was considered abnormal, while > 50% lumen diameter reduction was the criterion for significant CAD at CTCA.

**Results:**

Of 131 patients, 34 (26%) had ischemia at stress echo (new wall motion abnormalities), and 56 (43%) had reduced CFR on LAD. Significant coronary stenosis at CTCA was found in 69 (53%) patients. Forty-six patients (84%) with abnormal CFR on LAD showed significant CAD at CTCA (p < 0.001). Calcium score was higher in patients with reduced than in those with normal CFR (265 ± 404 vs 131 ± 336, p = 0.04). During a median follow-up of 7 months (1st to 3rd quartile: 5–13 months), there were 45 major cardiac events (4 deaths, 11 nonfatal myocardial infarctions, and 30 late [≥6 months] coronary revascularizations). At Cox analysis, independent prognostic indicators were calcium score > 100 (HR 2.84, 95% CI 1.33-6.07, p = 0.007), significant CAD at CTCA (HR 2.68, 95% CI 1.23-5.82, p = 0.013), and inducible ischemia or CFR <1.9R on LAD on dual imaging stress echo (HR 2.25, 95% CI 1.05-4.84, p = 0.038).

**Conclusions:**

Functional and anatomical evaluation using, respectively, dual imaging stress echocardiography and CTCA are both effective modalities to risk stratify patients with chest pain of unknown origin, yielding independent and comparable prognostic value. Compared to CTCA, however, stress echocardiography has the advantage of lower cost and of being free of radiations.

## Introduction

Dual imaging of wall motion and coronary flow reserve (CFR) in the left anterior descending coronary artery (LAD) is the current state-of-the-art technique for vasodilator stress echocardiography [1] providing superior diagnostic [2-7] and prognostic [8-10] value compared to standard stress testing. In particular, a reduced CFR in the LAD added prognostic information over wall motion analysis in patients with known or suspected coronary artery disease (CAD) [8]. Interestingly, the prognostic capability of CFR was not affected by ongoing anti-ischemic therapy [11,12].

Computed tomography coronary angiography (CTCA) has emerged as a noninvasive effective diagnostic imaging modality [13-16], providing good correlation with invasive measurement of stenosis severity [17]. In particular, CTCA demonstrated high accuracy and negative predictive value in patients referred for chest pain of unknown origin [17]. Unfortunately, CTCA provides a non-negligible radiation exposure with associated cancer risk [18,19]. In addition, it is unable to assess the hemodynamic meaning of vessel stenosis, as fewer than half of CTCA identified obstructive lesions confirmed by coronary angiography cause ischemia [20].

This prospective, observational study was aimed at assessing the prognostic value of dual imaging dipyridamole stress echocardiography and CTCA in a cohort of chest pain patients having suspected CAD.

## Methods

### Study patients

From 2008 to 2010, 138 chest pain patients were prospectively enrolled at the Cardiology Division, Umberto I Hospital, Mestre, fullfilling the following inclusion criteria: [1] stable chest pain syndrome; 2) no history of CAD (i.e., history of myocardial infarction, coronary revascularization, and/or angiographic evidence of > 50% diameter coronary stenosis); 3) adequate image quality allowing satisfactory wall motion analysis and LAD flow; 4) no significant valvular heart disease; 5) no co-morbidity reducing life expectancy to < 1 year. Of the 138 initially selected patients, 7 were lost to follow-up.

Thus, 131 patients (76 men; age 68 ± 9 years) formed the study group.

The study was approved by the institutional review board of Dell’Angelo Hospital Mestre-Venice Italy. All patients gave their written informed consent before undergoing stress echocardiography. When patients signed the written informed consent they also authorized physicians to use their clinical data according to Italian law. All patients were followed-up for a median of 9 months, with a minimum pre-defined follow-up time of 6 months.

### Resting and stress echocardiography

Transthoracic stress echocardiographic studies were performed with a commercially available ultrasound machine (iE 33, Philips Ultrasound, Andover, MA, USA) equipped with multifrequency phased-array sector scan probes (S3 to S8) and with second harmonic technology. Two-dimensional echocardiography and 12-lead electrocardiographic monitoring were performed in combination with high-dose dipyridamole (up to 0.84 mg over 6 min). Echocardiographic images were semiquantitatively assessed using a 17-segment, 4-point scale model of the left ventricle [1]. A wall motion score index was derived by dividing the sum of individual segment scores by the number of interpretable segments. Ischemia was defined as stress-induced new and/or worsening of pre-existing wall motion abnormality. Wall motion abnormality at rest was akinetic or dyskinetic myocardium with no thickening during stress. CFR was assessed during standard stress echocardiographic examination by intermittent imaging of wall motion and LAD flow [1]. Coronary flow in the mid-distal portion of the LAD was searched in the low parasternal long-axis section under the guidance of color Doppler flow mapping [1,2,7]. All studies were digitally stored to simplify off-line reviewing and measurements. Coronary flow parameters were analyzed off-line using the built-in calculation package of the ultrasound unit. Flow velocities were measured 2 times for each study, namely at baseline and at peak stress (before aminophylline injection). At each time point, three optimal profiles of peak diastolic Doppler flow velocities were measured, and the results were averaged. CFR was defined as the ratio between hyperemic peak and basal peak diastolic coronary flow velocities. Images were digitally stored at rest, at peak dipyridamole stress testing, and after aminophylline infusion [7]. A CFR on LAD < 1.9 was taken as abnormal [21]. Quality control of stress echocardiographic performance was previously described in depth [22]. Previously assessed intra- and interobserver variabilities for measurements of Doppler recordings and regional wall motion analysis assessment were < 10% [2].

### CT coronary angiography

Heart rate was controlled by intravenous beta blockers, usually metoprolol, administered before CTCA with a titration dose up to 20 mg in patients with heart rate 65 beats/min. In all patients, CTCA was performed using a 64-slice scanner (64 × 0.625 mm collimation, 330 ms gantry rotation time, VCT, GE Medical Systems, Milwaukee, WI, USA). Dose modulation was attained with “electrocardiographic gating” for a maximum gantry delivery between 40% and 80% during the R-R interval. A bolus of 80 ml of high-concentration contrast (Iomeron 400 mg/ml, Bracco Imaging, Milan, Italy) was administered intravenously at 5 ml/s, followed by 50 ml of saline injected at the same infusion rate. The scan was initiated according to the bolus-tracking technique. The coronary calcium score was assessed with dedicated software (CaScore Package, GE Healthcare, Milwaukee, WI, USA), and Agatston score was recorded.

A calcium score > 100 was considered significant [23-25].

Image datasets were analyzed using volume rendering and multiplanar reconstruction on post-processing workstations (CardioQ3 package, Advantage Workstation version 4.2, GE Healthcare) [23]. A semiquantitative scale was used by the CCTA readers to grade extent of luminal stenosis as a percentage of the vessel diameter using visual estimations.

A vessel stenosis ≥ 50% in at least one coronary artery was the criterion for CAD at CTCA. CTCA and dipyridamole stress echocardiography data were collected and analyzed by different observers. The imaging data were accessible to the referring physician (FR).

### Follow-up data

Outcome was determined from patient interviews at outpatient clinics, hospital chart reviews, and telephone interviews with a patient, a patient’s close relative, or referring physician. Clinical events recorded during follow-up were death, nonfatal myocardial infarction, early (<6 months) and late (≥6 months) coronary revascularization (surgery or angioplasty). To avoid misclassification of cause of death, overall mortality was considered. The diagnosis of acute myocardial infarction was made on the basis of symptoms, ECG changes, and cardiac enzyme level increases. Late revascularization was considered as clinical end-point, reflecting new or progressive symptoms. The data were analyzed for the prediction of major events (death, infarction, late revascularization).

### Statistical analysis

Data were expressed as mean ± standard deviation for continuous variables and as numbers (percent) for categorical variables. Continuous variables were compared using the Student’s unpaired t-test, while differences of categorical variables were assessed by the chi-square test. Linear regression was adopted to assess the correlation between calcium score and peak and rest-stress wall motion score index Kaplan-Meier curves were used for estimation of event rate. Only the first event was taken into account. Patients undergoing coronary revascularization were censored at the time of the procedure. The association of selected variables with outcome was assessed with the Cox proportional hazard model using univariate and stepwise multivariate procedures. A significance of 0.05 was required for a variable to be included in the multivariate model, and 0.1 was the cut-off value for exclusion. Hazard ratios with corresponding 95% confidence intervals were estimated. Statistical significance was set at p < 0.05.

SPSS 16.0 (SPSS, Inc., Chicago, IL, USA) was used for analysis.

## Results

The clinical and echocardiographic characteristics of the study group are shown in Table 1. Of 131 patients, 34 (26%) had ischemia at stress echocardiography, and 56 (43%) had impaired CFR on LAD. A significant CAD at CTCA was documented in 69 (53%) cases.

**Table 1:** **Baseline characteristics of the patients**

Age (*years*)	68 ± 9
Male gender (*%*)	83 (63%)
Smoke (*%*)	34 (26%)
Diabetes (*%*)	29 (27%)
Hypertension (*%*)	71 (54%)
Dyslipidemia (*%*)	77 (59%)
LV ejection fraction at rest (*%*)	56 ± 6
WMSI at rest	1.07 ± 0.16
Mean CFR on LAD (*cm/s*)	2.12 ± 0.58
TC calcium score	193 ± 373
Therapy	
*B-blockers*	58 (44%)
*Ca-antagonist*	30 (23%)
*Nitrates*	9 (7%)

Calcium score was higher in patients with reduced than in those with normal CFR on LAD (265 ± 404 vs 131 ± 336, p = 0.04). In particular, patients with reduced CFR on LAD showed higher percentage of calcium score >100, compared to those with normal CFR on LAD (32/55 pts, 58% vs 17/76 pts, 22%, p < 0.001).

In addition, calcium score significantly correlated withwall motion score index at peak stress (r = 0.222, p = 0.011) and rest-stress change in wall motion score index (r =0.225, p = 0.010). A calcium score >100 was observed in 25 of 34 patients with stress-induced ischemia and in 24 of 97 patients with no ischemia (74 vs 25%, p < 0.001).

CFR on LAD was also found to correlate with the presence of CAD at CTCA. Indeed, CAD at CTCA was evidenced in 46 of 55 patients with reduced CFR and in 26 of 76 patients with normal CFR (84 vs 34%, p < 0.001).

During a median follow-up of 7 months (1st to 3rd quartile: 5–13 months), 45 major cardiac events (4 deaths, 11 nonfatal myocardial infarctions, and 30 late coronary revascularizations) were registered. Additionally 19 patients underwent early revascularization and were excluded from outcome analysis.

**Figure 1:**
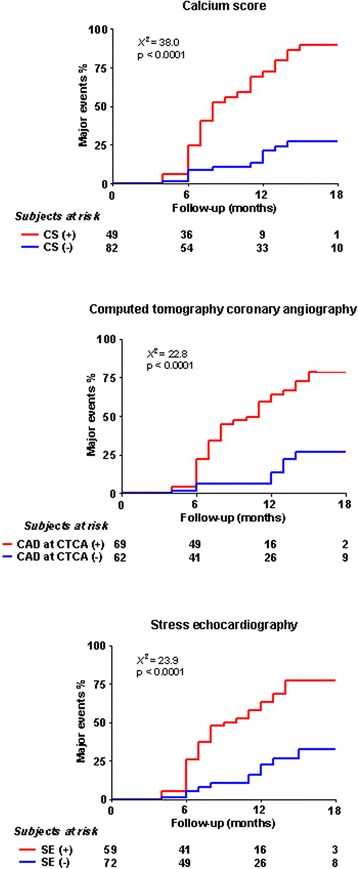
Kaplan-Meier survival curves for two different groups of the patients based on calcium score (Ca-score) ≥ 100 and Ca-score < 100 (Up).Kaplan-Meier survival curves for two different groups of the patients based on the presence (>50%) or absence of coronary artery stenosis at CTCA of at least one coronary artery (in the medium).Kaplan-Meier survival curves for the two different groups of patients based on abnormal CFR on LAD (≤1.9) and/or wall motion score index change positive for stress-induced ischemia compared to patients with normal CFR on LAD (>1.9) without stress-induced ischemia (Down).

At Cox analysis, calcium score > 100 (HR 2.84, 95% CI 1.33-6.07, p = 0.007), significant CAD at CTCA (HR 2.68, 95% CI 1.23-5.82, p = 0.013), and presence of inducible ischemia and/or reduced CFR on LAD (HR 2.25, 95% CI 1.05-4.84, p = 0.038) (Table 2) were multivariable prognostic indicators. The event rate was markedly (p < 0.0001) lower for patients with no ischemia and normal CFR on LAD, calcium score < 100, or no CAD at CTCA (Figure 1).

**Table 2:** **Univariate and multivariate predictors of events in the follow-up**

	**Univariate analysis**	**Multivariate analysis**		
	**HR (95% CI)**	***p***	**HR (95% CI)**	***p***
Age	1.059 (1.012-1.108)	*0.012*		
Gender (male)	1.640 (0.881-3.052)	*0.119*		
Smoking	0.733 (0.378-1.422)	*0.358*		
Diabetes	1.586 (0.850-2.959)	*0.147*		
Hypertension	0.75 (0.824-2.912)	*0.175*		
Hypercholesterol	1.122 (0.624-2.018)	*0.700*		
Family history	1.164 (0.888-3.035)	*0.144*		
Therapy	1.542 (0.796-2.987)	*0.199*		
Ejection fraction	1.010 (0.965-1.058)	*0.667*		
Rest WMSI	0.716 (0.131-3.914)	*0.700*		
CFR on LAD ≤ 1.9	3.095 (1.139-8.410)	*< 0.001*		
Stress-induced Ischemia	3.997 (2.144-7.558)	*0.005*		
Stress-induced Ischemia or CFR on LAD ≤ 1.9	4.475 (2.261-8.854)	*< 0.001*	2.252 (1.047-4.842)	*0.038*
Calcium score > 100	5.984 (3.088-11.599)	*< 0.001*	2.841 (1.329-6.075)	*0.007*
CAD at CTCA	4.911 (2.333-10.334)	*< 0.001*	2.681 (1.234-5.823)	*0.013*

CAD at CTCA: stenosis > 50% at least one coronary artery.

## Discussion

In patients with chest pain and suspected CAD, functional dual imaging-derived parameters such as inducible ischemia or reduced CFR on LAD and significant CAD at CTCA as well as vessel calcification provide strong and comparable prognostic information.

### Anatomic evaluation of coronary stenosis: CTCA

CTCA is a recognized modality for the non-invasive assessment of CAD, with a reported mean sensitivity and specificity >90% [26].

Calcium score adds prognostic value to that of traditional risk factors [23-25]. Of note, it allows to reclassify intermediate risk asymptomatic subjects in more accurate risk categories [27,28]. In this study we found a direct relationship between high calcium score and the presence of stress-induced ischemia or impaired CFR on LAD. Moreover, high calcium score was an independent predictor of future cardiac events.

Previous experiences on symptomatic unselected patient populations showed CTCA to be more accurate than calcium score to predict all-cause mortality [29-31]. However, both obstructive and non-obstructive CAD at CTCA was associated with increased mortality in the Confirm study [32]. In addition, in stable patients with suspected or known CAD, non-invasive fractional flow reserve computed from CT was associated with improved diagnostic accuracy and discrimination compared to CTCA alone for diagnosis of hemodynamically significant CAD [33].

### Functional evaluation of coronary stenosis: stress echocardiography

In recent years, dipyridamole stress echocardiography with combined assessment of CFR in the LAD has entered the echocardiography lab as a highly feasible technique [7], providing additional diagnostic value over conventional wall motion analysis [8-12,34]. In fact, dipyridamole stress echocardiography with combined assessment of CFR has also been extensively validated in its prognostic correlates and has allowed effective risk assessment in diabetics with negative stress echocardiographic results by wall motion criteria, [9], in subjects with intermediate LAD stenosis [10], and in those with normal or near-normal coronary arteries [11]. Interestingly enough, the prognostic capability of CFR was not affected by ongoing anti-ischemic therapy [12].

CFR on LAD or inducible wall motion abnormalities would certainly improve the cost-benefit practice compared with indiscriminate “carpet bombing” with dilatation for all stenosis independent of the underlying clinical picture and physiologic substrate. The American Heart Association/ American College of Cardiology/Society for Cardiovascular Angiography and Interventions on percutaneous coronary interventions guidelines and the American Heart Association scientific statement from the committee on diagnostic and interventional cardiac catheterization have indicated that in the presence of an intermediate lesions, when fractional flow reserve is < 0.75 or CFR is < 2.0, the stenosis is considered hemodynamically significant and percutaneous coronary intervention can be supported [35].

Accordingly, in this study we showed that CFR on LAD or stress-induced ischemia were independent predictors of events, and higher coronary calcification score was associated with lower values of CFR.

### Clinical use of the techniques

The clinical use of stress echo and CTCA should consider the cost, safety, effectiveness and feasibility issues that may vary in the various centers since - for instance - the radiation dose of CTCA may range from 1 to 30 mSv depending upon type of technology, operator skills, and clinical questions (calcium score vs coronary angiography) [18,19]. Stress echocardiography has several advantages, including the possibility of obtaining information on regional function and CFR in the same sitting, low cost, and radiation-free nature of the ultrasound technique. Yet, it is dependent upon the skill and experience of the reader and therefore not all centers have adequate expertise to guarantee high diagnostic standards [7-12,34].

### Study limitations

For uniformity of the study population, we enrolled all patients with symptoms (patients with chest pain syndrome of unknown origin) without history of previous myocardial infarction or previous myocardial revascularization. This represents a group with intermediate pre-test likelihood of disease, where the indication for noninvasive testing is more appropriate [35].

In all patients, medical therapy was kept unchanged. This may have caused suboptimal echo studies with false negative results for wall motion analysis. However, the prognostic capability of CFR on LAD is not affected by ongoing therapy [12].

## Conclusions

Dipyridamole stress echocardiography and CTCA are two noninvasive methods providing different and potentially complementary information on patients with chest pain and suspected CAD, giving excellent risk stratification. Both tests are valuable for risk stratification in patients with chest pain and suspected CAD, however stress echo is radiation free and has limited costs when compared to CTCA.
